# Magnitude, Determinants, and Coping Strategies of Food Insecurity Among People Living With HIV/AIDS in Eastern Ethiopia

**DOI:** 10.1155/arat/9970515

**Published:** 2025-04-19

**Authors:** Ararsa Demu, Aklilu Tamire, Negga Baraki, Abraham Negash, Mesay Dechasa, Jerman Dereje, Awoke Masrie, Samrawit Shawel, Abera Cheru, Tadesse Dufera, Abainash Tekola, Berhe Gebremichael

**Affiliations:** ^1^School of Public Health, College of Health and Medical Sciences, Haramaya University, Harar, Ethiopia; ^2^School of Nursing and Midwifery, College of Health and Medical Sciences, Haramaya University, Harar, Ethiopia; ^3^Department of Clinical Pharmacy, School of Pharmacy, College of Health and Medical Science, Haramaya University, Harar, Ethiopia; ^4^Department of Psychiatry, School of Nursing and Midwifery, College of Health and Medical Sciences, Haramaya University, Harar, Ethiopia; ^5^School of Environmental Science, College of Health and Medical Science, Haramaya University, Harar, Ethiopia

**Keywords:** coping strategies, determinant factors, East Ethiopia, food insecurity, HIV/AIDS

## Abstract

**Background:** Globally, over 2 billion people are affected by food insecurity linked to HIV/AIDS. In Africa, there are about 28.5 million people of all ages living with HIV/AIDS, of whom 2.2 million died of AIDS due to multiple factors that overlap, severe household food insecurity including inadequate food diversity, food intake less than three times a day, body mass index (BMI) of less than 18 kg per square meter, and inadequate food intake. Good nutrition is important for people with HIV because it helps strengthen the immune system and keeps people with HIV healthy and helps absorb HIV medicines. In sub-Saharan Africa, including Ethiopia, the high prevalence of starvation or famine exacerbated HIV/AIDS patients' mortality rates. National data from Ethiopia show that nearly 90% of HIV/AIDS-infected people are affected by food insecurity. The current study aimed to assess the magnitude, determinant factors, and coping strategies of food insecurity among adult people living with HIV/AIDS.

**Methods:** An institutional-based cross-sectional study was conducted on 421 adult HIV positives who were attending antiretroviral treatment at public health facilities in both rural and semiurban areas. The study subjects were selected by a simple random sampling technique. A pretested, semistructured questionnaire was used. A bivariate and multivariate logistic regression model was fitted to identify the independent factors associated with food insecurity. Adjusted odds ratio (AOR) with a 95% confidence interval (CI) was estimated to measure the strength of the association.

**Results:** Food insecurity was prevalent among 80.3% of HIV/AIDS patients in the study population. Living in a rural area, the presence of another family member with HIV, inadequate dietary diversity, low frequency of meals, and current high viral load in the last 12 months were some of the factors that significantly affect food insecurity among AIDS patients. Eating less preferred foods and reducing the number of meals were among common coping strategies.

**Conclusion:** This study analyzed the overall magnitude of food insecurity among HIV/AIDS patients and found it to be higher, which may end up in a shortening of life expectancy. A national health policymaker needs to integrate long-term food and nutrition interventions for marginalized groups, specifically PLWHA to tackle factors negatively affecting food insecurity and highly erosive coping strategies among AIDS patients.

## 1. Introduction

Food security exists when all people, at all times, have physical, social, and economic access to sufficient, safe, and nutritious food that meets their dietary needs and food preferences for an active and healthy life [1]. Globally, over 2 billion population were affected by food insecurity linked to human immunodeficiency virus/acquired immune deficiency syndrome (HIV/AIDS) disease in both resource-rich and resource-poor settings [2]. It was estimated that there were 37.6 million people living with HIV/AIDS (PLWHA), 1.5 million newly infected, 27.4 million receiving antiretroviral therapy (ART), and 690 who died from HIV-related causes at the end of 2020 [3].

HIV/AIDS are worldwide public health issues that worsen food insecurity in households by lowering work capability and productivity in the most economically and productive age group of society. Evidence suggests that HIV infection itself impairs nutritional status, reduces food security, and causes weight loss. These factors are linked to lower survival rates among individuals living with HIV because they result in poor nutrient absorption, decreased food intake, and altered body utilization of nutrients that are either stored or received [4–6].

Food insecurity and HIV/AIDS are correlated in both directions, with food insecurity contributing to poor nutritional status and raising the chance of HIV infection. However, by decreasing agricultural output, income, hospitalization, and medical costs, HIV infection might result in food insecurity and a diminished ability to address the food crisis [6, 7]. As nutrient needs increase after HIV infection, reducing the amount and quality of food consumed by PLWHA may be a coping mechanism [7].

About 28.5 million individuals of all ages in Africa are living with HIV/AIDS, and 2.2 million of them have died from the disease as a result of a number of factors that coexist with severe household food insecurity. These factors include inadequate food diversity, eating less than three times a day, having a body mass index (BMI) of less than 18 kg per square meter, and inadequate food intake [8]. Extreme malnutrition among PLWHA can result from this acute food insecurity, which is linked to negative consequences associated with HIV/AIDS [9]. Food insecurity can be both endemic and epidemic among PLWHA, and it can exacerbate clinical and health consequences like treatment default, despair, anxiety, and mortality. Severe malnutrition among PLWHA can be caused by food insecurity, and the most opportunistic infection that can arise from severe malnutrition is tuberculosis [8].

In sub-Saharan Africa, the prevalence of starvation or famine highly exacerbated the HIV/AIDS patients' mortality rate [10]. In this region out of the total of PLWHA, 40% of them faced household food insecurity [11]. Specially, in poor resource settings, food insecurity is highly widespread, which might impede immunological reconstitution and increase patient mortality in the early ART period. The elevated risk of mortality among PLWHA is due to malfunction of the immune system that results from inadequate diet [12].

In Ethiopia, there were an estimated 36,990 new HIV infections for all ages [13]. About 10% of the general population and 40.4%–87.4% of PLWHA struggle to obtain “safe, sufficient, and nutritious food” for themselves and their families, making this country one of the many sub-Saharan African nations that are severely impacted by food insecurity [14]. In Benishangul-Gumuz regional state of Ethiopia, the prevalence of household food insecurity among PLWHA was 76% [15], while in the southern part of this country the prevalence of food insecurity was 57.3% [16].

As coping means of the household's food insecurity, many people use their assets like eating less preferred, less expensive, and begging for foods [17, 18]. People typically seek loans from family members and neighbors during food crises, buy food and rely on assistance from others, gather wild foods and immature crops, eat the seed from the next planting season, encourage family members to eat meals prepared by friends or relatives, limit daily meal portions, eat only once a day, skip meals to feed their children, go days without eating, take farmland as a contract from others, and migrate to neighboring regions or countries in search of wage employment [13]. There were few research studies on the prevalence of food insecurity, its determinants, and coping mechanisms among PLWHA undergoing ART follow-up at Eastern Ethiopia's public health facilities, despite reports of some levels of food insecurity in the general community. The study methodology and the results of this study were taken from the original master's thesis [19].

## 2. Methods and Materials [19]

### 2.1. Study Area and Period

The study was conducted at public health facilities of West Hararghe Zone, both rural and semiurban areas of Oromia Regional State, Eastern Ethiopia. This study area is composed of 92% rural area and 8% urban area. The zone has 15 rural woredas, two towns, and 493 kebeles. The area has more than three million people, of whom 2950 are PLWHA.

On the other hand, the zone has six hospitals and 83 health centers. From these, 18 health facilities have ART clinics. This study was conducted from May 15 to June 15, 2022. An institutional-based cross-sectional study design was conducted on PLWHA who were on follow-up and receiving ART at public health facilities.

### 2.2. Sample Size Determination

The sample size of the study was calculated using the single population proportion of 0.533 (0.533%) magnitude of food insecurity taken from the previous study [20] and assumption of margin of error (d) = 5%, Zα/2 = 1.96 or 95% confidence interval

(1) $$\begin{array}{c} n={\left(Z\frac{\alpha }{2}\right)}^{2}\frac{p\left(1-P\right)}{d^{2}},\\ n={\left(1.96\right)}^{2}\frac{0.533\left(1-0.533\right)}{{\left(0.05\right)}^{2}},\\ n=3.84\frac{0.248911}{0.0025}=382.3. \end{array}$$

By adding 10% contingency, the final sample size became 421.

### 2.3. Sampling Techniques and Data Collection Tool Development

**Figure 1:**
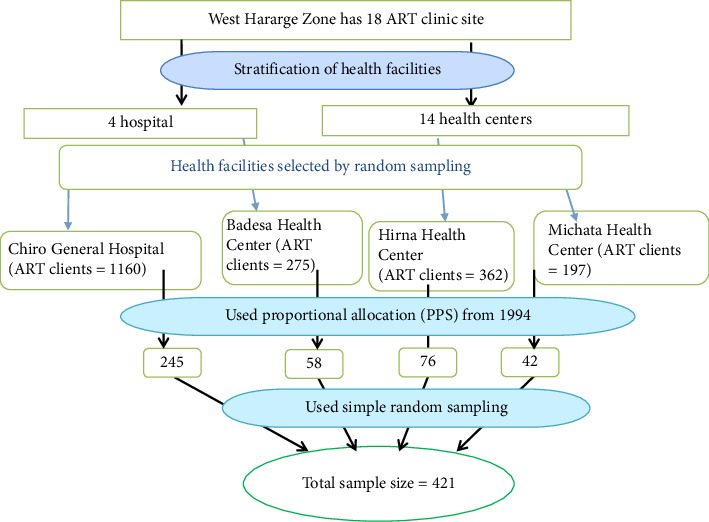
Schematic presentation of the sampling procedure of study participants of food insecurity and coping strategies among people on ART follow-up, Eastern Ethiopia [19].

A stratified sampling method was used to identify study subjects. Health facilities were stratified into hospitals and health centers. Then, from the stratum, one hospital and three health centers were selected by simple random sampling techniques. The number of patients participating from each health facility was allocated by probability proportional to size (Figure 1). The study participants were selected randomly using a lottery method based on patients' ART unique identification number (MRN).

### 2.4. Data Collection Procedures

A structured questionnaire was developed from previous works [21, 22]. All questionnaires were translated into local language and then translated back to English by language experts. Data collection tools consist of six parts. The first part was sociodemographic-related questionnaires. The second part was the questionnaires related to household dietary diversity (HDD) which was also developed by using previous work as a baseline [23] with a total of 12 food items that measure household food insecurity in terms of the varieties of nutrients eaten by household members within the previous 24 hours.

The second part was the Household Food Insecurity Access Scale (HFIAS) that was extracted from the previous study [24]. This was originally developed by Food and Nutrition Technical Assistance (FANTA) and measures food insecurity in terms of 18 question items. These 18 items have two components. The first 9 items were occurrence questions that were responded to in terms of no = 0 and yes = 1 or which have a minimum score of 0 to a maximum score of 9. The second 9 items were frequency questions derived from occurrence questions with the response option of yes.

### 2.5. Data Collection Procedure

The data were collected by face-to-face interview technique using semistructured questionnaires. To measure the weight, the client was requested to remove the shoe and wear a light dress. Data collectors weighed the study participants on the portable digital scale, and the value was recorded to the nearest kilogram. To measure the height, the client was requested to stand erect with their shoulders level, hands at the side, and their head, scapula, buttock, calf, and heel in contact with the vertical measuring board. The height was measured to the meter using a height-measuring device with a sliding head bar following standard anthropometric techniques [25].

### 2.6. Study Variables [19]

#### 2.6.1. Dependent Variables

Food insecurity was considered a dependent variable.

#### 2.6.2. Independent Variables

Sociodemographic and economic factors included age, sex, family size, number of children, educational status, ethnicity, religion, residence, marital status, head of household, other person living with HIV, monthly household income, and occupation status.

### 2.7. Data Quality Control

To assure the quality of the data, a structured and pretested questionnaire was used. A pretest was conducted on 5% (21 HIV-positive clients on ART) of the sample size at separate health center that was outside the actual study setting before the actual data collection period. Data collectors check and assure the appropriateness and functionality of digital weight scales using known weights every morning before the data collection process begins. Questionnaires were checked to ensure whether they are correctly filled or not. Double data entry was performed to realize consistency in data entry and to correct mismatches by cross-checking. In addition, the quality of data collection was ensured through close supervision of the data collectors.

### 2.8. Data Analysis [19]

After the completion of the data collection process, all the data were checked for completeness and consistency. Then, the data were cleaned, edited, coded, and entered into Epi Data Version 3.1 and exported to SPSS Windows Version 25 for analysis. Descriptive statistics, including percentage, proportion, and frequency, were used to describe the data. To identify the factors associated with food insecurity, both bivariable and multivariable logistic regression analyses were conducted consecutively. First, the dependent variable was dichotomized into “1” for the presence of food insecurity and “0” for its absence. Then, a bivariable logistic regression model was fit to see the crude association between each independent and dependent variable. Both crude and adjusted odds ratios with 95% confidence intervals were estimated to show the presence, strength, and direction of associations. Statistical significance was declared at a *p* value of 0.05.

## 3. Results [19]

### 3.1. Sociodemographic Characteristics

Of 421 study participants, 275 (65.3%) were female and 36.3% were in the age group of 26–35 years with the mean (±SD) age of 37.92 (±9.19) years, with a minimum age of 19 years and maximum age of 80 years. Two hundred seventy-seven (65.8%) participants were from male-headed households. Two hundred seventy-two (64.6%) were urban dwellers. The majority (58.4%) were married. On the other hand, 156 (37%) were daily labors, 172 (40.9%) had completed primary school education, and 316 (75%) of the participants' families earned less than 2500 ETB per month (Table 1).

**Figure 2:**
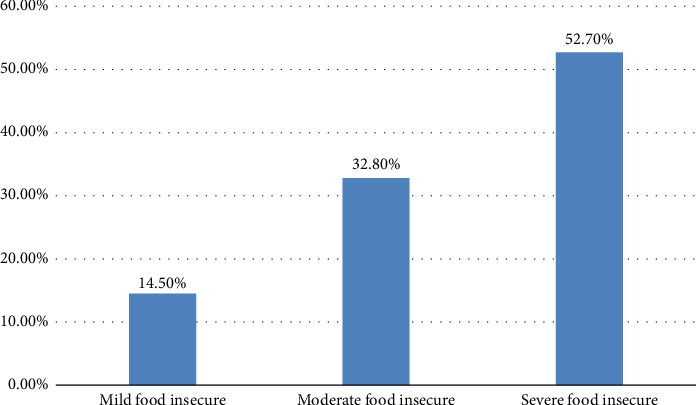
Level of food insecurity among adult people living with HIV/AIDS on follow-ups at public health facility of Hararge, Ethiopia. Dietary diversity, meal frequency, and nutritional status among adults on ART in public health facilities of Eastern Ethiopia (*n* = 421).

The overall proportion of food insecurity among PLWHA receiving ART at public health facilities of Hararge, Ethiopia, was 338 (80.3%). The level of food insecurity among participants was classified as mild 29 (6.89%), moderate 106 (25.18%), and severe 203 (48.22%) food insecurity (Figure 2).

The majority (332 [78.9%]) of adults on ART were worried about food, while (329 [78.1%]) were unable to eat preferred food. On the other hand, 192 (45.6%) of adults on ART had no any kind of food in the household (Table 2).

### 3.2. Coping Strategies in Response to Food Insecurity

In response to food insecurity, the majority (308 [91%]) of PLWHA eat less preferred food. Furthermore, 216 (64%) and 203 (60%) of the PLWHA sell household assets and eat limited portions of meals, respectively (Table 3).

### 3.3. Prevalence of Household Food Insecurity and Its Determinant Factors

Those living in rural areas were 3.3 times more likely to be food insecure than those living in urban areas (AOR = 3.3; 95% CI: 1.6, 6.7). The presence of other persons living with HIV in the family was two times more likely to be food insecure than the absence of other persons living with HIV (AOR = 2.1; 95% CI 1.2–3.6). Current viral load in the last 12 months or less (cp/mL) ≥ 1000 was 2.3 times (AOR = 2.3 95% CI 1.1–4.8) food insecure than < 1000 cp/mL (Table 4).

## 4. Discussion

The current study evaluated coping mechanisms, determining factors, and the extent of food insecurity among ART-treated HIV/AIDS patients. The percentage of PLWHA getting ART who experienced food insecurity was 338 or 80.3%. Food insecurity was influenced by a number of characteristics, including residing in an urban or rural area, having another HIV-positive family member, having a current viral load of less than 12 months, and consuming a diet that is not diverse enough. Less than three meals a day were among the factors that had the biggest impact on food insecurity. However, this study found that PLWHA adopt the following coping mechanisms to deal with food.

Similar to previous studies [6, 26], about 80% of research participants experienced food insecurity. However, it was higher than the findings of research conducted in Ethiopia and other African nations [5, 6, 27, 28]. Nevertheless, this result is lower than related study conducted in Fiche zonal hospital and Debre Markos, Ethiopia [29, 30]. The variation in magnitude among different parts of the countries could be due to the existence of differences in socioeconomic status, health intervention measurement, study setting, study methodology, and nutritional policy among HIV/AIDS patients.

Since food insecurity among PLWHA typically increases HIV transmission, inaccessibility to HIV treatment, decreased ART adherence, lower baseline CD4 cell count, virology control, and increased mortality, incorporating food security interventions into HIV/AIDS treatment is essential to stopping the epidemic and improving the health and quality of life of the infected population [31]. However, food insecurity also causes poor pregnancy outcomes, sadness, a sense of helplessness, shame, and hazardous sexual conduct as a coping mechanism [32].

Rural communities were more likely to be food insecure than those living in urban areas which was similar to other studies even though the strength of the association was different [5, 26]. Lower socioeconomic status, less access to and variety of food, a lack of infrastructure services in rural areas (only natural resource bases), low input, crop-based systems, knowledge and information systems about health and education, marketing and credit systems, and limited access to social services and health services in rural areas relative to urban settings could all be contributing factors to the negative relationship between food insecurity and living in a rural area [33]. People who live in rural areas and are at risk of starvation typically eat seeds that will be sown in successive harvest years as a coping mechanism or survival tactic that can reduce the possibility of crops [34, 35]. Additionally, among PLWHA in rural areas, food insecurity led to increased rates of hunger, disease, inflation, high unproductive force, food shortages, poor mental health, anxiety, and depression, as well as more infectious and noncommunicable diseases [36].

Likewise, the lack of dietary diversity has an impact on food insecurity, which is consistent with research conducted in other contexts. [6, 26]. If people cannot obtain enough food, in terms of both quality and quantity, to meet the needs of household members, food insecurity may worsen. This may have to do with a lack of knowledge about dietary diversity as well as their inaccessibility and lack of availability in the market and research area. Undiversified food consumption resulted in premature deaths from infectious, noncommunicable, and hunger diseases [37].

According to the current study, food insecurity is impacted by PLWHA's low daily meal frequency, which is also consistent with the findings of other studies [38, 39]. According to recommendations, PLHA should eat three meals or more a day due to their higher nutritional needs [1]. Despite these suggestions, a sizable percentage of research participants said they ate fewer than three meals a day, suggesting that many of them were working to reduce their current level of food insecurity. This further implies that information regarding underweight resulting from fewer meals and illness processes should be tracked during PLWHA screening. Research conducted elsewhere has demonstrated that PLWHA in environments with limited resources frequently lack access to necessary foods, making it difficult for them to meet recommended dietary intakes [20, 39].

Food insecurity was more common among participants who had family members with HIV/AIDS than among those who did not. This finding is in line with the prior research [40], which suggests that there may be more HIV/AIDS-positive individuals living in a single family, which could result in higher medical expenses, more missed workdays from illness, and food poverty. When one or more members of the household get ill with HIV/AIDS, food insecurity begins to occur as the productive forces begin to decline due to this HIV/AIDS-related disease. Members of the household experience food insecurity when the productive forces are reduced, which can worsen opportunistic illnesses like HIV/AIDS. Additionally, there can be increasing healthcare costs and demands, which could affect food insecurity [40].

Food insecurity was more common among research participants with a high viral load (> 1000 cp/mL) than among those with a suppressed viral load (< 1000 cp/mL). This may be explained by the fact that food instability is linked to lower productive forces the greater the viral load, which can further impair immunity and raise the likelihood of another related illness. Poor adherence, physical weakness, and decreased productivity are some of the negative clinical effects that can result from this for PLWHA. These include a decline in physical health status, a decrease in CD4 count, a rise in the incidence of serious illness, and an increase in visits to medical facilities as a result of frequent illness.

The majority of dietary coping mechanisms found in this study were significantly worsened by opportunistic illnesses and HIV/AIDS. The nutritional condition of the PLWHA and the other household members is negatively impacted by the majority of the different diets and nondietary coping mechanisms that have been identified as being employed by households amid food shortages [6, 7, 41]. As dietary needs increase after HIV infection, PLWHA may find that reducing the amount and quality of food they consume is a highly erosive coping mechanism. While some techniques are simple to recover from, others, such as sex for money and moving to an urban region for work, are more challenging and can worsen food insecurity and HIV/AIDS transmission.

## 5. Conclusion

This study analyzed the overall magnitude of food insecurity among PLWHA and found it to be higher, which may end up in a shortening of life expectancy. Rural residents, the presence of other family members with HIV, low frequency of meals, inadequate dietary diversity, and high viral load of 1000 cp/mL were the factors affecting food insecurity among PLWHA. Most of the erosive coping strategies lead to other associated problems, like more HIV/AIDS transmission among the community, malnutrition, more poverty as people usually sell their household assets, and overlap with many nutritional problems. A national health policymaker needs to integrate food and nutrition interventions for marginalized groups, specifically PLWHA.

## Figures and Tables

**Table 1:** Sociodemographic characteristics of the patients on ART at public health facilities of West Hararghe, Ethiopia.

Variables	Category	Frequency	Percent
Sex	Male	146	34.7
	Female	275	65.3

Age in years	18–25	30	7.1
	26–35	153	36.3
	36–44	146	34.7
	> 45	92	21.9

Sex of head of household	Male	277	65.8
	Female	144	34.2

Residence	Urban	272	64.6
	Rural	149	35.4

Religion	Orthodox	242	57.5
	Muslim	165	39.2
	Protestant	14	3.3

Marital status	Married	246	58.4
	Divorced	115	27.3
	Widowed	38	9
	Single	22	5.2

Educational status	Unable to read and write	100	23.8
	Able to read and write	65	15.4
	Only primary education (1–8th)	172	40.9
	Secondary education (9–12th)	63	15
	College diploma and above	21	5

Occupation	Farmer	46	10.9
	Housewife	32	7.6
	Merchant	110	26
	Government employee	31	7.4
	Daily labor	156	37
	Other	44	10.4

Living condition	Alone	38	9
	With family/spouse	361	85.7
	With parent (mother/father)	22	5.2

Family size	≤ 5	308	73.2
	> 5	113	26.8

Average monthly income	> 2500	105	24.9
	1001–2500	159	37.8
	≤ 1000	157	37.3

**Table 2:** Adults on ART patients Household Food Insecurity Access Scale (HFIAS) in public health facilities, Hararge, Ethiopia, 2022 (*n* = 421).

HFIAS question	Yes		No	
	Frequency	Percent	Frequency	Percent
Worry about food	332	78.9	89	21.1
Unable to eat preferred foods	329	78.1	92	21.9
Eat limited variety of foods	329	78.1	92	21.9
Eat foods that did not want to eat	317	75.3	104	24.7
Eat smaller meal	293	69.6	128	30.4
Reduced the amount of meal	234	55.6	187	44.4
Go to sleep at night hungry	140	33.3	281	66.7
No food in kind in the household	192	45.6	229	54.4
Go whole day and night without eating anything	61	14.5	360	85.5

**Table 3:** Coping strategies in response to food insecurity among adults on ART patients in public health facilities, West Hararghe, Eastern Ethiopia (*n* = 338).

Coping strategy	Frequency	Percent
Eating less preferred food	308	91
Eating limited portion size	203	60
Reducing the number of meals	216	64
Purchase food by credit	89	26
Borrowing money to buy food	73	22
Selling household assets	74	22
Sending children to work	54	16
Doing hard work	47	14
Begging	46	13.6
Migrate to urban areas for wage	39	15
Pity trading	31	9
Sex for money	22	6.5

**Table 4:** Factors associated with food insecurity among adults on ART patients in public health facilities, West Hararge, Eastern Ethiopia.

Variables	Categories	Food security status		COR (95% CI)	AOR (95% CI)	*p* values
		Food secure	Food insecure			
Sex	Male	37 (25.4)	109 (74.6)	1	1	
	Female	46 (16.7)	229 (83.3)	1.6 (1.03, 2.7)	1.2 (0.7, 2.2)	0.414

Sex of head of household	Male	70 (25.3)	207 (74.7)	1		
	Female	13 (9)	131 (91)	3.4 (1.8, 6.4)	1.2 (0.2, 6.5)	0.052

Residence	Urban	72 (26.5)	200 (73.5)	1	1	
	Rural	11 (7.4)	138 (92.6)	4.5 (2.3, 8.8)	3.3 (1.6, 6.7)^∗^	0.001

Marital status	Married	59 (24)	187 (76)	1	1	
	Divorced	16 (14)	99 (86)	3.1 (0.7, 13.8)	4.0 (0.8, 20.0)	0.082
	Widowed	6 (16)	32 (84)	1.6 (0.3, 7.5)	2.9 (0.5, 16.0)	0.216
	Single	2 (9)	20 (91)	1.8 (0.3, 10.2)	4.8 (0.7, 32.7)	0.111

Family size	≤ 5	69 (22.4)	239 (77.6)	1	1	
	> 5	14 (12.4)	99 (87.6)	2.0 (1.1, 3.7)	1.8 (0.9, 3.6)	0.883

Presence of other persons living with HIV in the family	No	41 (26.3)	115 (73.7)	1	1	
	Yes	42 (15.8)	223 (84.2)	1.8 (1.1, 3.0)	2.1 (1.2, 3.6)^∗^	0.007

Current viral load in the last 12 months or less (cp/mL)	< 1000	72 (22.3)	251 (77.7)	1	1	
	≥ 1000	11 (11.2)	87 (88.8)	2.2 (1.1, 4.4)	2.3 (1.1, 4.8)^∗^	0.021

Current khat chewing	Yes	11 (8.6)	117 (91.4)	3.4 (1.7, 6.7)	0.8 (0.1, 4.3)	0.805
	No	72 (24.6)	221 (75.4)	1	1	

Dietary diversity	Inadequate (< 4 types)	20 (9.2)	198 (90.8)	4.4 (2.5, 7.7)	3.7 (2.0, 6.6)^∗^	≤ 0.001
	Adequate (≥ 4 types)	63 (31)	140 (69)	1	1	

Meal frequency	< 3 times	15 (8.9)	154 (91.1)	3.7 (2.0, 6.9)	3.3 (1.7, 6.3)^∗^	≤ 0.001
	≥ 3 times	68 (27)	184 (73)	1	1	

^∗^Indicates: *p* value is significant at 0.05.

## Data Availability

The data that support the findings of this study are available on request from the corresponding author. The data are not publicly available due to privacy or ethical restrictions.

## Nomenclature

AIDS: Acquired immune deficiency syndrome
ART: Antiretroviral therapy
BMI: Body mass index
ETB: Ethiopian Birr
HIV: Human immunodeficiency virus
